# The “ART” of Linkage: Pre-Treatment Loss to Care after HIV Diagnosis at Two PEPFAR Sites in Durban, South Africa

**DOI:** 10.1371/journal.pone.0009538

**Published:** 2010-03-04

**Authors:** Elena Losina, Ingrid V. Bassett, Janet Giddy, Senica Chetty, Susan Regan, Rochelle P. Walensky, Douglas Ross, Callie A. Scott, Lauren M. Uhler, Jeffrey N. Katz, Helga Holst, Kenneth A. Freedberg

**Affiliations:** 1 Division of General Medicine, Massachusetts General Hospital, Boston, Massachusetts, United States of America; 2 Division of Infectious Disease, Massachusetts General Hospital, Boston, Massachusetts, United States of America; 3 Department of Medicine, Brigham and Women's Hospital, Boston, Massachusetts, United States of America; 4 Department of Orthopedics, Brigham and Women's Hospital, Boston, Massachusetts, United States of America; 5 Division of Rheumatology, Brigham and Women's Hospital, Boston, Massachusetts, United States of America; 6 Departments of Biostatistics and Epidemiology, Boston University School of Public Health, Boston, Massachusetts, United States of America; 7 Center for AIDS Research, Harvard Medical School, Boston, Massachusetts, United States of America; 8 Department of Medicine, Harvard Medical School, Boston, Massachusetts, United States of America; 9 Departments of Epidemiology and Health Policy and Management, Harvard School of Public Health, Boston, Massachusetts, United States of America; 10 Department of Medicine, McCord Hospital, Durban, South Africa; 11 Department of Medicine, St. Mary's Hospital, Durban, South Africa; Instituto de Medicina Tropical Alexander Von Humboldt, Peru

## Abstract

**Background:**

Although loss to follow-up after antiretroviral therapy (ART) initiation is increasingly recognized, little is known about pre-treatment losses to care (PTLC) after an initial positive HIV test. Our objective was to determine PTLC in newly identified HIV-infected individuals in South Africa.

**Methodology/Principal Findings:**

We assembled the South African Test, Identify and Link (STIAL) Cohort of persons presenting for HIV testing at two sites offering HIV and CD4 count testing and HIV care in Durban, South Africa. We defined PTLC as failure to have a CD4 count within 8 weeks of HIV diagnosis. We performed multivariate analysis to identify factors associated with PTLC. From November 2006 to May 2007, of 712 persons who underwent HIV testing and received their test result, 454 (64%) were HIV-positive. Of those, 206 (45%) had PTLC. Infected patients were significantly more likely to have PTLC if they lived ≥10 kilometers from the testing center (RR = 1.37; 95% CI: 1.11–1.71), had a history of tuberculosis treatment (RR = 1.26; 95% CI: 1.00–1.58), or were referred for testing by a health care provider rather than self-referred (RR = 1.61; 95% CI: 1.22–2.13). Patients with one, two or three of these risks for PTLC were 1.88, 2.50 and 3.84 times more likely to have PTLC compared to those with no risk factors.

**Conclusions/Significance:**

Nearly half of HIV-infected persons at two high prevalence sites in Durban, South Africa, failed to have CD4 counts following HIV diagnosis. These high rates of pre-treatment loss to care highlight the urgent need to improve rates of linkage to HIV care after an initial positive HIV test.

## Introduction

While the AIDS epidemic continues to devastate sub-Saharan Africa, where it remains the leading cause of death [1], substantial progress has been made in efforts to provide HIV care and treatment to those in need. In South Africa, antiretroviral therapy (ART) rollout began in 2004, and by December 2006, ART had reached 32% of those eligible for treatment [2]. The South African Department of Health made access to care a top priority in its National Strategic Plan for HIV/AIDS that was unveiled in March 2007 [3]. In addition to reducing the number of new infections, the primary aim of this plan is expanding access to treatment and support services to reach 80% of HIV-infected people in need [3].

Maximal effectiveness of ART depends on timely HIV diagnosis, linkage to care, treatment initiation, and retention in care. The success of ART programs in South Africa depends in large part on the ability to identify HIV-infected individuals, to determine ART eligibility and monitor those not yet eligible for ART to facilitate timely transition to treatment, to initiate care among those eligible, and to ensure sustainable access to care over time. High rates of loss to follow-up (LTFU) following ART initiation have been reported from many sites [4]–[6]. A recent review of ART programs in sub-Saharan Africa found rates of LTFU ranging from 20% at 6 months to nearly 40% at 2 years after ART initiation [6]. While these studies describe the rates of LTFU after ART initiation, there is a paucity of data on loss to care after HIV diagnosis, but prior to initiating ART. Our objective was to examine the rates of pre-treatment loss to care (PTLC) and to determine factors associated with a higher likelihood of PTLC for newly diagnosed HIV-infected persons.

## Methods

### Ethics Statement

We obtained written, informed consent from all study participants. The protocol, questionnaires, and consent forms were approved by the McCord Hospital Ethics Committee, the Ethics Committee of St. Mary's Hospital Mariannhill, and the Partners HealthCare Human Research Committee in Boston, MA, USA.

### Setting

We conducted a prospective cohort study in two sites in KwaZulu-Natal, the province with the highest HIV prevalence in South Africa [1]. McCord Hospital, located in Durban, is a Christian, 142-bed state-aided (public/private partnership) general hospital serving a predominantly urban population from the greater Durban area, as well as more distant parts of KwaZulu-Natal. McCord charges subsidized fees for services and treatment. ART has been available since 2001, but prior to the President's Emergency Plan for AIDS Relief (PEPFAR)-funded scale-up of ART in 2004, patients had to pay the full cost of their treatment, which averaged 900-1500 Rand per month ($125-$210 US, 2007), because of the high cost of ARV drugs. Gross national income per capita in South Africa was $5,730 US in 2007 [7]. Since PEPFAR implementation in 2004, HIV-infected patients pay a subsidized, inclusive monthly fee of 140 Rand ($20 US, 2007), which covers all clinic visits, laboratory testing, and medications. Currently over 10,000 people have enrolled in the HIV program at McCord, of which over 6,800 have begun ART. Throughout this report, McCord Hospital is referred to as the ‘urban hospital.’

St. Mary's Hospital Mariannhill, located 20 kilometers west of Durban, is a Catholic, state-aided, 200-bed general district hospital serving a poorer population from a defined catchment area in the surrounding communities. St. Mary's Hospital serves primarily to the healthcare needs of the “poorest of the poor” living in rural and peri-urban areas of the Durban Metropolitan area. A higher state subsidy enables St. Mary's to charge lower fees for all services than McCord. The iThemba Family Care Centre, based at St. Mary's, began offering ART in February 2003, and is free of charge to patients. Since then, over 3,900 people have had access to ART there. In this report we refer to St. Mary's as the ‘rural hospital.’

Comprehensive care services at both hospitals include outpatient medical care, social work, psychological services, pastoral care, and training and income generation projects for support group members. Both hospitals have been awarded PEPFAR funding to expand the number of patients on ART. Other sources of funding include the KwaZulu-Natal Provincial Department of Health and subsidized patient fees for McCord Hospital and the Department of Health and hospital-raised funds for St. Mary's Hospital. Together these sites have more patients on treatment and more years of experience in treating patients on ART than any other sites in the KwaZulu-Natal, South Africa public sector.

### HIV Testing Protocols

The urban hospital offered HIV testing, counseling, and referral, using rapid HIV tests. Patients eligible for the study were offered testing in the outpatient department. Patients generally received HIV test results within 30 minutes of testing. Those with a positive initial rapid HIV test underwent confirmatory testing with a rapid HIV test from a second manufacturer. Patients diagnosed with HIV were referred to the HIV clinic in an adjacent building, approximately 300 meters away. There, CD4 testing via venipuncture was offered to patients who registered and paid the clinic registration fee of 90 Rand ($12 US, 2007). Venipuncture specimens were sent to the National Health Laboratory Service or Global Laboratories (both located in Durban) for flow cytometry.

The rural hospital's protocol for HIV testing was based on a venipuncture for a standard ELISA (Centaur XP, Siemens Healthcare Diagnostics, Deerfield, IL, USA or Roche E170, Roche, Basel, Switzerland). Patients eligible for the study were offered HIV testing at the primary health care clinic. Patients were asked to return for HIV test results within two weeks of HIV testing. Positive results were confirmed with a Western Blot. CD4 testing for HIV-infected patients was offered at the same site free of charge to the patients immediately after they received an HIV-positive test result. Venipuncture specimens were sent to the National Health Laboratory Service or Lancet Laboratories (both located in Durban) for flow cytometry, and patients had to return for the results after one month.

### Cohort Overview

All English or Zulu speaking adults (≥18 years old) who presented for HIV testing at each of the study hospitals between November 2006 and May 2007 were eligible for enrollment in the South African Test, Identify and Link (STIAL) Cohort, provided they were able to give informed consent, were not pregnant, did not present to care in a stretcher or wheelchair, were not already known to be HIV-infected, and were willing to share HIV test results with research staff. Those enrolled were asked to fill out a voluntary baseline questionnaire, offered in English or Zulu by trained study personnel. For study participants who were newly identified as HIV-infected at the study visit, medical record review was done within 12 weeks of study enrollment to document receipt of HIV test results, presence of follow-up for CD4 count, and results of the CD4 count test.

### Data Elements

Patient level data were collected in the following four domains:

1. *Demographic characteristics* included age, gender, marital status, primary language spoken at home, educational attainment, employment status, and household composition.
2. *Geographic characteristics* included self-reported distance between the participant's residence and the health center, time spent traveling from home to the center, and mode of transportation to reach the clinic.
3. *Clinical characteristics* were self-reported and included reasons for visiting the hospital, means of referral for HIV testing, history of tuberculosis (TB), frequency of using health care services during the prior 6 months and self-rated health. Self-rated health was ascertained using one question asking about rating a person's health between ‘very good’, ‘good’, ‘fair’ and ‘poor’. The ‘excellent’ category from a standard 5-point self-rating of health was omitted due to the lack of differentiation in Zulu between ‘excellent’ and ‘very good’.
4. *Laboratory information* obtained from clinic records included dates and results of HIV test and CD4 count for those who had CD4 tests.

### Statistical Analysis

#### 1) Study outcomes

The primary outcome of the study—pre-treatment loss to care (PTLC)—was defined by the authors as failure to undergo a CD4 count within 8 weeks of receiving an HIV-positive test result. Thus, PTLC was calculated as the number of newly diagnosed HIV-infected study participants who did not return for a CD4 test within 8 weeks of HIV diagnosis divided by the total number of newly diagnosed HIV-infected study participants.

#### 2) Analysis

Bi-variate and multivariate analyses were performed to determine the independent predictors of PTLC. Variables exhibiting association with outcomes at a bi-variate risk ratio (RR) of ≥1.5 or ≤0.60 or at a p-value of <0.10 were advanced into multivariate models to control for confounding and to estimate the independent impact of each factor on PTLC. Log-linear models were utilized for multivariate analysis to estimate risk ratios (RR), since PTLC was not a rare outcome and odds ratios would not accurately estimate risk ratios. We implemented a two-level model building strategy. First, we built models within each of 3 domains: clinical, demographic, and geographic. Then, we developed a series of hierarchical models (demographic, demographic+geographic, and demographic+geographic+clinical) to determine the most parsimonious overall model.

Because patient factors associated with a higher likelihood of PTLC may be inter-correlated, we combined them into a single measure which we termed the ‘index of vulnerability for pre-treatment loss to care.’ This measure was calculated as the sum of the following characteristics, which emerged as predictors of PTLC in multivariate analysis: ≥10 km distance between patient residence and site of care, a history of treatment for TB, and referral for HIV test by a medical care provider as opposed to self-referral. The ‘index of vulnerability’ values could range from 0 (no risk factors) to 3 (all 3 risk factors). We estimated a dose-response relationship between the index of vulnerability and PTLC by calculating a p-value for linear trend. We also estimated the risk ratio of PTLC for persons with various numbers of risk factors compared to persons who had none of the risk factors. All analyses were carried out using Stata statistical software (Stata Statistical Software Release 9, StataCorp, College Station, TX, USA).

## Results

### Cohort Characteristics

**Figure 1:**
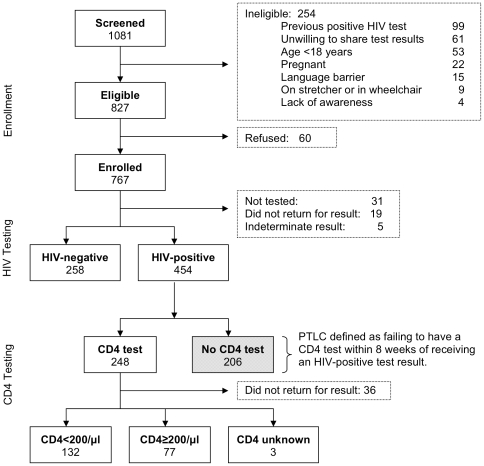
Study enrollment, HIV testing and CD4 testing flow chart. PTLC: Pre-treatment loss to care.

Among 1,081 persons screened for the study between November 2006 and May 2007 at the two hospitals, 827 were eligible, and 767 (93%) of those eligible enrolled. The percent eligible among those screened did not differ by site (76% at the rural hospital vs. 77% at the urban hospital), but those eligible were more likely to enroll at the rural hospital than at the urban hospital (97% vs. 90%, p<0.001). The most common reasons for ineligibility were having had a previous positive HIV test, unwillingness to share test results with research staff, and age <18 years (Figure 1). Almost all enrollees indicated Zulu as their primary language. Eighty percent of enrollees were <45 years of age and 54% were male (Table 1). These characteristics were similarly distributed among study participants from both centers. Overall, 25% of study participants had primary school education or no formal education and 34% had completed *matric* (equivalent to completing high school in the US school system) or had received higher education. The educational level varied between the sites, with 30% of study participants at the urban hospital having had primary education or less compared to 18% of study participants at the rural hospital. Seventy-two percent of study participants enrolled at the rural hospital reported living ≥10 km from the center compared to 45% of study participants from the urban hospital.

**Table 1:** Site-stratified and overall characteristics of a cohort of persons presenting for HIV testing in two PEPFAR sites, Durban, South Africa.

Factor	Urban		Rural		Overall	
	N (%)^a^	HIV-pos. (%)^b^	N (%)^a^	HIV-pos. (%)^b^	N (%)^a^	HIV-pos. (%)^b^
**Total**	382 (100)	211 (55)	330 (100)	243 (74)	712 (100)	454 (64)
**Demographic characteristics**						
Age (years)						
Under 45	299 (78)	176 (59)	267 (82)	205 (77)	566 (80)	381 (67)
45 and over	83 (22)	35 (42)	58 (18)	34 (59)	141 (20)	69 (49)
Sex*						
Male	193 (51)	113 (59)	195 (59)	142 (73)	388 (54)	255 (66)
Female	189 (49)	98 (52)	135 (41)	101 (75)	324 (46)	199 (61)
Education**						
Primary or none	112 (30)	61 (54)	60 (18)	43 (72)	172 (25)	104 (60)
Some high school	108 (29)	70 (65)	184 (56)	139 (76)	292 (42)	209 (72)
Matric^c^ or more	154 (41)	76 (49)	83 (25)	58 (70)	237 (34)	134 (57)
Employment status						
No full-time job	195 (52)	104 (53)	190 (58)	141 (74)	385 (55)	245 (64)
Full-time job	183 (48)	105 (57)	137 (42)	100 (73)	320 (45)	205 (64)
Marital/living arrangement**						
Unmarried/Lives alone	42 (12)	26 (62)	51 (16)	37 (73)	93 (14)	63 (68)
Unmarried/Lives with others	204 (60)	131 (64)	224 (69)	170 (76)	428 (64)	301 (70)

Factor	Urban		Rural		Overall	
	N (%)^a^	HIV-pos. (%)^b^	N (%)^a^	HIV-pos. (%)^b^	N (%)^a^	HIV-pos. (%)^b^
Married/With children	83 (24)	29 (35)	30 (9)	18 (60)	113 (17)	47 (42)
Married/No children	12 (4)	5 (42)	19 (6)	14 (74)	31 (5)	19 (61)
**Geographic characteristics**						
Distance from home to facility**						
<10 kilometers	211 (55)	111 (53)	90 (28)	54 (60)	301 (43)	165 (55)
≥10 kilometers	171 (45)	100 (58)	232 (72)	185 (80)	403 (57)	285 (71)
**Clinical characteristics**						
TB treatment history**						
Yes	34 (9)	22 (65)	63 (19)	50 (79)	97 (14)	72 (74)
No	347 (91)	189 (54)	267 (81)	193 (72)	614 (86)	382 (62)
Reason for HIV testing**						
Referred by health care provider	322 (85)	181 (56)	139 (42)	103 (74)	461 (65)	284 (62)
Self- or other referral	57 (15)	28 (49)	191 (58)	140 (73)	248 (35)	168 (68)
Self-rating of health**						
Very good	8 (2)	3 (38)	20 (6)	8 (40)	28 (4)	11 (39)
Good	237 (64)	115 (49)	123 (38)	80 (65)	360 (52)	195 (54)
Fair	117 (32)	82 (70)	124 (38)	96 (77)	241 (35)	178 (74)
Poor/bad	7 (2)	5 (71)	60 (18)	57 (95)	67 (10)	62 (93)

a % of total at hospital or overall.

b % of total in category.

c Equivalent to completing high school in the US school system.

*denotes p-values 0.01<p<0.05 in comparing characteristics across two sites.

**denotes p-values p<0.001 in comparing characteristics across two sites.

### HIV Prevalence

Of 712 enrolled patients who were tested and received conclusive results, 454 were HIV-infected, for an overall HIV prevalence of 64% (95% CI: 60%-67%). HIV prevalence was significantly higher among study subjects enrolled at the rural hospital, with 243 of 330 enrollees HIV-infected (74%; 95% CI: 69–78%) compared to subjects enrolled at the urban center, with 211 of 382 enrollees HIV-infected (55%; 95% CI: 50%–60%). HIV prevalence was similar between men and women, and no significant association was found between prevalence of HIV and level of education, employment status, or marital status. Among study participants reporting a history of being treated for TB (9% at the urban hospital vs. 19% at the rural hospital; 14% overall), the prevalence of HIV was 65% in the urban center and 79% in the rural center (74% overall). One hundred thirty-two (62%) of those receiving CD4 counts and their results had CD4 counts <200/µl (68% at the urban hospital and 60% at the rural hospital, p = 0.238; Figure 1).

### Factors Associated with Pre-Treatment Loss to Care (PTLC)

Nearly half of the 454 newly diagnosed HIV-infected persons (206; 45%) had PTLC (Table 2). In multivariate analysis, patients who lived ≥10 km from the center (RR = 1.37; 95% CI: 1.11–1.71), patients with a history of being treated for TB (RR = 1.26; 95% CI: 1.00–1.58), and patients referred for HIV testing by a health care provider rather than self-referred (RR = 1.61; 95% CI: 1.22–2.13) were more likely have PTLC. Notably, an additional 36 patients (15%) who had a CD4 count within 8 weeks did not return for the results of that CD4 count (Figure 1).

**Table 2:** Factors associated with pre-treatment loss to care after HIV diagnosis in a cohort of newly diagnosed HIV-infected persons in two PEPFAR sites, Durban, South Africa, 2006–2007.

Factor	N (total)	% PTLC	Crude RR (95% CI)	Adjusted RR (95% CI)
**Total**	454	45.4	—	—
**Demographic characteristics**				
Age (years)				
45 and over	69	36.2	1.00	1.00
Under 45	381	46.7	1.29 (0.93, 1.80)	1.30 (0.95, 1.78)
Sex				
Female	199	43.2	1.00	1.00
Male	255	47.1	1.09 (0.89, 1.34)	1.13 (0.93, 1.38)
Education				
Primary or none	104	43.3	1.00	
Some high school	209	45.9	1.06 (0.81, 1.38)	
Matric^a^ or more	134	45.5	1.05 (0.79, 1.40)	
Employment status				
No full-time job	245	43.3	1.00	
Full-time job	205	47.8	1.10 (0.90, 1.35)	
Marital/living arrangement				
Unmarried/Lives alone	63	46.0	1.00	
Unmarried/Lives with others	301	45.9	1.00 (0.74, 1.34)	
Married/With children	47	51.1	1.11 (0.75,1.63)	
Married/No children	19	36.8	0.80 (0.42, 1.53)	

Factor	N (total)	% PTLC	Crude RR (95% CI)	Adjusted RR (95% CI)
**Geographic characteristics**				
Distance from home to facility				
<10 kilometers	165	40.6	1.00	1.00
≥10 kilometers	285	48.8	1.20 (0.96, 1.50)	1.37 (1.11, 1.71)
Site of care				
Rural hospital	243	37.0	1.00	1.00
Urban hospital	211	55.0	1.48 (1.21, 1.82)	1.40 (1.12, 1.77)
**Clinical characteristics**				
TB treatment history				
No	382	44.0	1.00	1.00
Yes	72	52.8	1.20 (0.94, 1.54)	1.26 (1.00, 1.58)
Reason for testing				
Self- or other referral	168	31.0	1.00	1.00
Referred by health care provider	284	54.2	1.75 (1.36, 2.25)	1.61 (1.22, 2.13)
Self-rating of health				
Very good	11	54.6	1.00	
Good	195	47.2	0.86 (0.49, 1.51)	
Fair	178	43.3	0.79 (0.45, 1.40)	
Poor/bad	62	41.9	0.77 (0.42, 1.42)	

RR: risk ratio.

CI: confidence interval.

a Equivalent to completing high school in the US school system.

**Figure 2:**
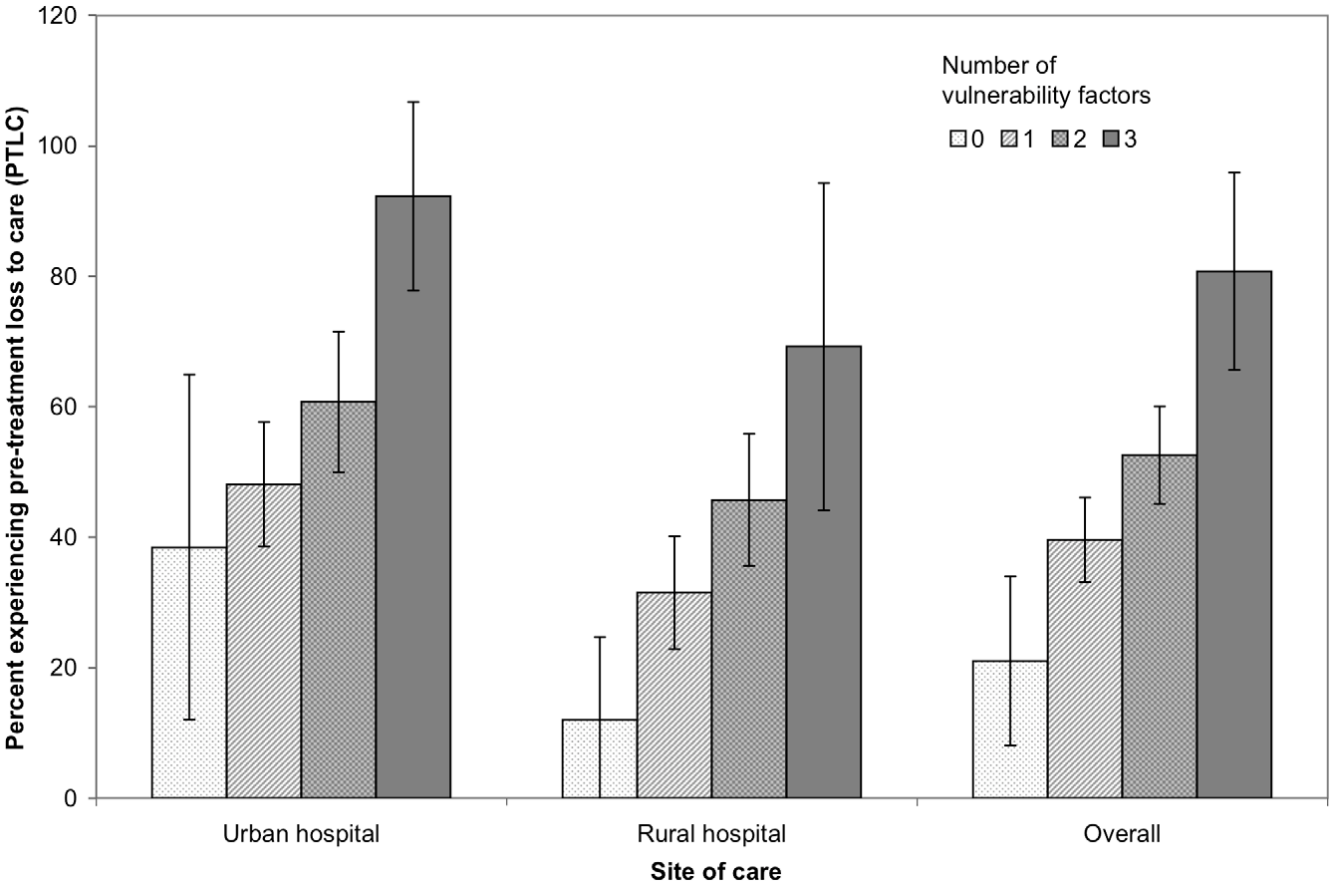
Likelihood of pre-treatment loss to care following an initial HIV-positive test, Durban, South Africa, 2006–2007.

The distribution of the index of vulnerability for PTLC was similar across the two hospitals. Five percent of all HIV-infected persons had all three risk factors (≥10 km from the center, history of TB treatment, and referral by a medical care provider), 36% had two risk factors, 48% had one risk factor, and only 10% had none of these risk factors. HIV-infected study participants with one, two and three of these risk factors for PTLC were 1.88, 2.50 and 3.84 times more likely to have PTLC compared to study participants with no risk factors (p-value for linear trend <0.0001). A greater number of risk factors led to a higher likelihood of PTLC in both the urban and the rural center, as well as the two centers combined (Figure 2).

## Discussion

In a population-based cohort presenting for HIV testing at two South African HIV outpatient sites, we found that nearly half of newly diagnosed HIV-infected persons had pre-treatment loss to care (PTLC) as defined by not following up with a CD4 count. Factors associated with higher rates of PTLC were: living ≥10 km from the health center, a history of TB treatment, and referral for HIV testing by a health care provider as opposed to self-referral. However, even among patients with none of these risk factors, a high proportion (21%) were still lost to care before initiating treatment. Among those who did return for a CD4 count, 62% had advanced HIV disease (CD4 count <200/µl).

We have found a small number of published studies that have examined loss to care prior to starting ART among patients newly diagnosed with HIV infection in resource-scarce settings [8], [9]. Although risk factors identified for loss to follow-up (LTFU) from studies in Africa include male sex [10] and lack of community support [11], the current study may not be directly comparable to studies on LTFU, since there may be different reasons for loss to care prior to and after initiation of treatment. Loss to care prior to ART initiation could be due to a number of causes, including stigma and denial of HIV diagnosis, perceived need for repeat testing, transfer to another clinic, or death.

Several studies in Africa have tracked patients who failed to keep clinic appointments and ascertained their reasons for nonattendance. Detailed tracking has revealed high rates of mortality among those considered lost, with 27–59% mortality in patients whose status could be determined [5], [12]–[15]. Of those still alive who could be reached, the most commonly reported reasons for nonattendance at clinic appointments were transfer to another clinic [5], [13], [16], [17] as well as financial reasons, including the high cost of transportation and the cost of the visit itself [12], [16], [18]. The current study, showing a greater risk of PTLC for patients living ≥10 km from the hospital, supports findings from studies that have tracked missing patients and found that the logistics of getting to a distant clinic are a major reason why patients fail to return to their site of care [12], [13], [18]. Following receipt of their HIV-positive test result, patients may have chosen to pursue HIV care at a center other than one of the study sites. There are approximately 20 alternative accredited ART initiation centers in the greater Durban area, with a denser distribution around the urban hospital. The greater number of alternative locations to initiate HIV care may contribute to the higher rate of PTLC we observed at the urban hospital.

Previous research has also shown that cost to the patient is a deterrent to continuing in HIV care [12], [16], [18]. Our findings are consistent with these studies, as we saw higher rates of PTLC when a fee for CD4 testing was charged at the urban center, compared to the rural center, which did not impose charges for CD4 testing. The differential rates of PTLC persisted between centers, even after adjusting for differences in demographic, clinical, or geographic factors; the difference may be due to structural features at the clinics, such as the cost of the CD4 test. In a recent review of retention in ART programs in sub-Saharan Africa, programs that did not require any payment from patients had higher retention rates at six months compared to those requiring partial or full payment [6]. Furthermore, investigators from the ART-LINC Collaboration examining mortality after one year in 18 ART programs in low-income countries found that access to treatment free of charge to the patient was associated with lower mortality [4].

Our finding that patients self-referred for HIV testing have lower rates of PTLC compared to patients referred by health care providers suggest that patients referring themselves for an HIV test are likely more motivated to follow-up with subsequent care. Those referred by physicians may need time to come to terms with the diagnosis and are therefore less prepared to deal with the consequences of knowing their HIV-positive status in the short term. These findings suggest that physicians referring patients for HIV testing should consider complementing that referral with the social support needed to make it effective. In addition to possibly being less motivated, patients referred for HIV testing by a physician may be sicker than self-referred patients, and therefore more likely to have died or been hospitalized at another site. Similarly, patients with a history of TB treatment may have had greater PTLC because they were more immune-compromised than other patients, and may have died or been too sick to return to the care center.

This study has several limitations. We relied on several self-reported measures, including distance from site of care and history of TB treatment. While the findings of this study may apply to settings similar to the two sites in Durban, it may not be possible to generalize the findings to all of South Africa, or to other resource-limited settings. We also used data for patient CD4 follow up at the site of the HIV test for only 8 weeks following receipt of a positive HIV test result. If patients returned after more than 8 weeks, or had a CD4 count done at another site, those results were not reflected in this study.

Despite these limitations, this study also has several methodological strengths. The first lies in the unique nature of the cohort: enrollment was offered at the time of HIV testing, prior to knowledge of HIV status, and was therefore not biased by the HIV test results. We enrolled patients consecutively, minimizing selection bias. Data were collected by study staff not involved in the care of patients. Finally, the study personnel recruiting patients were bilingual in English and Zulu, ensuring clear communication between study participants and staff.

The major finding of this study is that nearly half of newly diagnosed HIV-infected patients at two sites in South Africa had pre-treatment loss to care; they failed to obtain a CD4 count within 8 weeks after initial HIV diagnosis. Since a CD4 count is the first step toward linkage to effective HIV care and toward determining eligibility for ART, these findings—and appropriate remedies—are critical to ensure the success of HIV treatment scale-up efforts in South Africa. Of those who did obtain a CD4 count and result, nearly two-thirds had advanced HIV disease (CD4<200/µl) and thus met guidelines for beginning antiretroviral therapy. This high proportion of patients presenting with advanced disease highlights the urgent need to identify HIV-infected patients earlier in the course of their illness. One recent study from South Africa reported 34% mortality in patients lost to follow up prior to ART initiation [19]. Another recent South African study found rates of pre-treatment mortality of over 30 per 100 person-years and early treatment mortality rates of almost 20 per 100 person-years [17].

Understanding reasons for pre-treatment loss to care is critical for the development of targeted interventions to increase the number of people who access and remain in HIV care. This significant rate of loss to care warrants the expansion of adherence and retention efforts focused on those starting ART to all persons from the time they receive an initial HIV-positive test result. Strategies to increase pre-treatment linkage to care might include providing reimbursement for transportation to the clinic, providing CD4 testing free of charge to the patient, and contacting patients with reminders about appointments and test results. The substantial loss of patients after their initial HIV-positive test result, and prior to initiating HIV care, highlights the urgent need for strategies to be developed to improve successful linkage to care and retention in South Africa.
